# The prelimbic cortex but not the anterior cingulate cortex plays an important role in social recognition and social investigation in mice

**DOI:** 10.1371/journal.pone.0284666

**Published:** 2023-04-21

**Authors:** Joi Yashima, Tomoko Uekita, Toshiro Sakamoto

**Affiliations:** Department of Psychology, Graduate School of Health Sciences, Faculty of Health Sciences, Kyoto Tachibana University, Kyoto, Japan; University of Queensland, AUSTRALIA

## Abstract

The prefrontal cortex (PFC) has been implicated in social cognitive functions and emotional behaviors in rodents. Each subregion (prelimbic cortex, PL; infralimbic cortex; and anterior cingulate cortex, ACC) of the PFC appears to play a different role in social and emotional behaviors. However, previous investigations have produced inconsistent data, and few previous studies directly compared the roles of the PFC subregions using the same experimental paradigm. Accordingly, in the present study, we examined the role of the PL and the ACC in short-term social recognition, social investigation, and anxiety-related behaviors in C57BL/6J mice. We subjected mice with a lesioned PL or ACC, as well as those in a sham control group, to tests of social recognition and social novelty where juvenile and adult male mice were used as social stimuli. In the social recognition test, the PL-lesioned mice exhibited habituation but not dishabituation regardless of whether they encountered juvenile or adult mice. In a subsequent social novelty test, they spent less time engaged in social investigation compared with the control mice when adult mice were used as social stimuli. These results suggest that PL lesions impaired both social recognition and social investigation. In contrast, ACC-lesioned mice did not exhibit impaired short-term social recognition or social investigation regardless of the social stimulus. Furthermore, PL lesions and ACC lesions did not affect anxiety-related behavior in the open field test or light-dark transition test. Our findings demonstrate that the PL but not the ACC plays an important role in social recognition and social investigation.

## Introduction

The prefrontal cortex (PFC) plays an essential role in social cognitive function and emotional behaviors in rodents and primates, and dysfunction of this area has been linked to several psychiatric disorders such as depression, schizophrenia, and autism spectrum disorders in humans [1–4]. The PFC has several subregions; the prelimbic cortex (PL), infralimbic cortex (IL), and anterior cingulate cortex (ACC) [1], which appear to play different roles in social and emotional behaviors [1, 5–13]. However, evidence regarding the specific contributions of the subregions is scarce and inconsistent, and few previous studies directly compared the roles of the PFC subregions using the same experimental paradigm. Accordingly, in the present study, we examined the roles of the PL and ACC in social recognition, social investigation toward novel conspecifics, and anxiety-related behaviors in mice.

Social recognition, which refers to the ability to recognize other individuals and to discriminate between familiar and novel conspecifics, has been examined using the habituation-dishabituation paradigm and the social discrimination paradigm [14–17]. Recently, the PFC was reported to be important for social recognition memory [18, 19] in addition to the hippocampus [18, 20–27], amygdala [18, 28], nucleus accumbens (NAc) [29, 30], and insular cortex [31]. The involvement of the PFC and its subregions in social recognition memory differs depending on the memory type. For instance, long-term social recognition memory (longer than 1 day) requires protein synthesis at the PL and the ACC [18], and was found to be impaired by a PFC (PL, IL, and ACC) lesion [19]. In contrast, short-term social recognition memory (within 1–2 hours) was not impaired by a PFC (PL, IL, and ACC) lesion [19], but was impaired by an ACC lesion [6]. Thus, previous data on the involvement of the PFC and its subregions in short-term recognition memory are contradictory. In addition, the role of the PL in short-term social recognition has not been examined. To address this in the present study, we subjected mice with PL or ACC lesions, performed via microinjection of ibotenic acid, to a short-term social recognition test (SRT) [32].

Social recognition is strongly affected by social investigation, and both can be measured via social approach behavior [32, 33]. Therefore, the effects of lesions on social recognition should be examined according to alterations in social investigation. Social investigation can be assessed by measuring the duration of approach, exploratory, and interaction behavior toward a social stimulus in the social interaction test and three-chamber test [1, 15, 34, 35]. The PFC has been suggested to regulate social investigation [7, 34] involving the hippocampus [36], amygdala [7, 34, 36], NAc [37, 38], and insular cortex [39]. Each subregion of the PFC appears to have a different function with respect to social investigation. For instance, PL lesions were found to increase social investigation in mice [5], while ACC lesions decreased social investigation in rats [6]. In this study, we used the social novelty test (SNT), which uses four novel stimuli, to examine the roles of the PL and ACC in social investigation [40].

Social recognition and social investigation can be modulated by the type of social stimuli, for example, whether mice used as stimuli are juveniles or adults. In a previous study, PFC-lesioned and sham control mice exhibited a lower duration of social approach behaviors toward adult male mice compared with juvenile mice in the SRT and three-chamber sociability test [19]. Therefore, we used adult male mice as test mice, and juvenile and adult male mice as social stimuli in both the SRT and SNT based on our previous study [19].

The PFC plays an important role in emotional behaviors, especially anxiety-related behaviors. Although the PL and the ACC, which have neural connections to the basolateral amygdala (BLA) [7, 34, 41, 42], are involved in the modulation of anxiety-related behaviors, data regarding the involvement of the PFC in anxiety-related behaviors in rodents is inconsistent [9, 34]. Therefore, we conducted open-field and light-dark transition tests to examine the roles of the PL and ACC in general activity and anxiety-related behaviors.

The purpose of the present study was to examine the roles of the PL and ACC in short-term social recognition and social investigation regarding juvenile or adult male conspecifics, as well as anxiety-related behaviors, in mice. We performed four behavioral tests: the SRT, SNT, open field test, and light-dark transition test in PL- or ACC-lesioned mice.

## Material and methods

### Animals

Male C57BL/6J mice were obtained from a commercial breeder (Japan SLC, Inc., Hamamatsu, Japan). A total of 90 test mice aged 6 weeks were group-housed (2–4 mice/cage) in clear plastic cages (23 × 33 cm, 14 cm in height) containing wood shavings as bedding. Juvenile (3 weeks old: *n* = 32) and adult male mice (the same age as the test mice: *n* = 32), singly housed in small plastic cages (12 × 20 cm, 11 cm in height), were used as stimuli for the SRT and SNT. All animals were housed in an environment maintained at a temperature of 23 ± 1˚C and a humidity of 50 ± 10% with a 12-hour light/dark cycle (lights on at 7:00 a.m.). All animals had *ad libitum* access to food and water in their home cages. All experiments and animal care were conducted in compliance with Japanese law and the Kyoto Tachibana University Guidelines for Animal Experiments. All protocols were approved by the Ethics Committee for Animal Experiments of Kyoto Tachibana University (code: 20–1, 21–4).

### Surgery for pharmacological lesions

The surgical procedures for PL or ACC lesions were conducted according to a previous study [19]. At 7 weeks of age, the test mice were anesthetized via 0.3 mg/kg of medetomidine, 4.0 mg/kg of midazolam, and 5.0 mg/kg of butorphanol and mounted on a stereotaxic apparatus (David Kopf Instruments, Tujunga, CA, USA). A midline incision was made down the scalp and the area of bone above the injection sites was removed using a dental drill. The mice in the lesion group received a bilateral injection of ibotenic acid solution (dose: 10 μg/μL [43]) into the PL or ACC. The stereotaxic coordinates referred to a previous study [PL: +2.75 mm AP, ±0.3 mm ML, -1.55 mm DV from bregma [5]; volume: 0.15 μL/side, ACC: +1.1 mm AP, ±0.4 mm ML, -2.2 mm DV from bregma [44]; volume: 0.20 μL/side] and the mouse brain atlas [45]. The ibotenic acid solution was administered over 1 minute using a microsyringe (10 μL, 26s gage; Hamilton Company, Rino, Nevada, USA) with a microsyringe pump (Legato 130, KDScientific Inc., Holliston, Massachusetts, USA). The needle was kept in place for 1 minute after administration. The mice in the sham control group underwent the same surgery except for the drug injection. The scalp incision was sutured after removing the microsyringe. After a one-week recovery period, the mice underwent behavioral tests.

### Behavioral tests

The test mice were subjected to four behavioral tests (the SRT, SNT, open-field test, and light-dark transition test) in that sequential order at 8–9 weeks of age. The order of the test mice within each behavioral test was counterbalanced in the sham and lesioned groups. All behavioral tests were performed in the light phase. The experimental room was dimly lit (25 lx) for all tests.

#### Social recognition test (SRT) and social novelty test (SNT)

A white plastic box (36 × 31 cm, 17 cm in height) was used in both the SRT and SNT. The brightness in the box was 15 lx. A transparent Plexiglas cylinder (7 cm in bottom diameter, 5 cm in top diameter, 17 cm in height) with 28 holes (12 mm in diameter) on the lower side (SSI-MCGL, O’Hara & Co., Ltd. Tokyo, Japan) was used to present the stimulus mouse.

**Fig 1:**
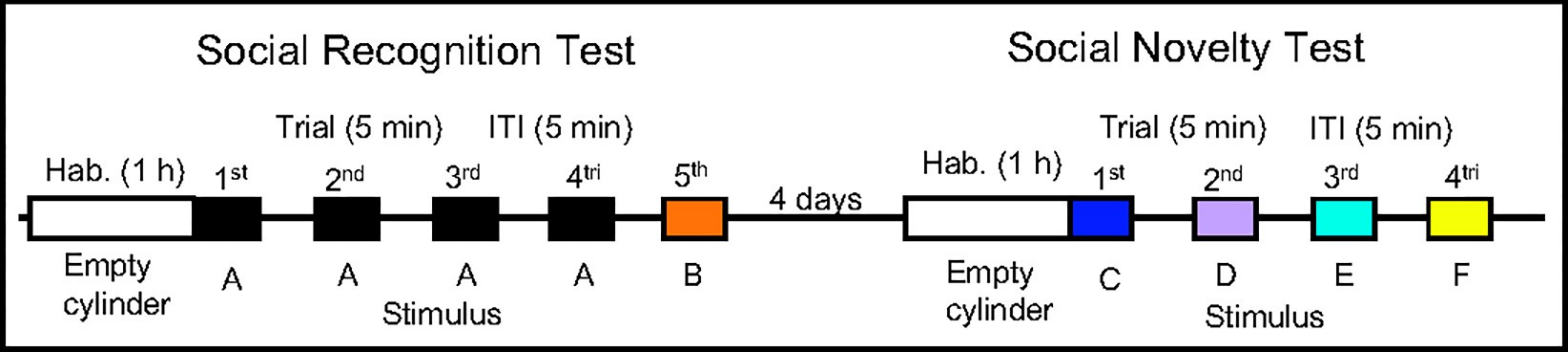
The timeline of the social recognition test (five trials) and the social novelty test (four trials). Both tests were performed after a 1-h habituation period, and all trials were 5 min long [ITI: inter-trial intervals].

Fig 1 shows the timeline of the SRT and SNT. During the habituation period (1 hour), an empty cylinder was placed in the center of the box, which was filled with fresh bedding, and the test mouse was allowed to freely explore the box. The SRT was performed using the habituation-dishabituation paradigm, as per previous studies [32, 40]. We conducted five SRT trials. In each trial, a stimulus mouse was placed in the cylinder at the center of the box, and the behavior of the test mouse was monitored. Each trial lasted 5 min, with 5-min inter-trial intervals. The same stimulus mouse was presented in the 1st–4th trial and a novel stimulus mouse was presented in the 5th trial. The SNT was performed four days after the SRT. After a habituation period (1 hour), the SNT began. We conducted four SNT trials. In each trial, a different stimulus mouse was placed in the cylinder at the center of the box and the behavior of the test mouse was monitored. Each trial lasted 5 min with 5-min inter-trial intervals. The behavior of the test mouse was recorded by a camera positioned above the box. Social investigation (SI) behavior was defined as active sniffing toward the stimulus mouse in the cylinder, and the SI duration and total traveled distance for each test mouse was measured using an analysis program (TimeSSI, O’Hara & Co, Ltd. Tokyo, Japan) on a Windows computer connected to the camera.

The stimulus mice were either juveniles or adults in both the SRT and SNT. The SRT and SNT were performed for the following eight pairings of a test mouse and a stimulus mouse: PL lesion group-juvenile (*n* = 10), sham group-juvenile (*n* = 11), PL lesion group-adult (*n* = 11), sham group-adult (*n* = 11), ACC lesion group-juvenile (*n* = 13), sham group-juvenile (*n* = 12), ACC lesion group-adult (*n* = 11), and sham group-adult (*n* = 11).

### Open field test

A wooden open field box (45 × 60 cm, 23 cm in height) with the bottom covered in white vinyl sheeting was used in the open field test [19]. A test mouse was placed at the corner of the open field and allowed to freely explore the open field for 10 minutes. The box was wiped clean with 70% alcohol after each test. The behavior of each test mouse was recorded by a camera positioned above the box. The total traveled distance and the time spent in the center area (27 cm × 40 cm) were measured using an analysis program (TimeOFC1 software, O’Hara & Co, Ltd.) on a Windows computer connected to the camera.

#### Light-dark transition test

A light-dark box containing a white and black box (41 × 20 cm, 25 cm in height, LD3002D, O’Hara & Co., Ltd.) connected by a small doorway (5 × 3 cm) was used in the light-dark transition test [19]. The white box was brightly lit with LED lights (400 lx), while the black box was kept dark (0 lx). At the beginning of the test, a test mouse was placed in the dark box and allowed to freely explore the light-dark box for 10 minutes. The boxes were wiped clean with 70% alcohol after each test. The behavior of the test mouse was recorded using cameras positioned on the ceiling of each box. The time spent in the light box and the latency to the first entry into the light box were measured for each mouse using an analysis program (TimeLD4 software, O’Hara & Co., Ltd.) on a Windows computer connected to the cameras. The light-dark transition test was performed on approximately half of the test mice in the four groups (PL lesion; *n* = 10, ACC lesion; *n* = 13, each sham group; *n* = 11, *n* = 12, respectively).

### Histological methods

After all of the behavioral tests were complete, the test mice were transcranially perfused with ALTFiX (Falma, Co., Ltd., Tokyo, Japan) under deep anesthesia. The brain was removed and fixed with ALTFiX for at least one day at 4 ˚C. Coronal sections (40 μm) were cut and stained with cresyl violet. The location and extent of the injury lesion were confirmed under a microscope. The investigators who conducted the histological analyses were blinded in terms of the behavioral results.

### Data analysis

We used the anovakun function (ver. 4.8.5.) in R software (ver. 4.2.0, R Core Team, Vienna, Austria) for statistical analysis. We used a three-way repeated-measures analysis of variance (ANOVA) to assess the group differences SRT and SNT scores. The simple effects test and the Bonferroni test were used for *post hoc* analysis. Student’s *t*-tests were used to compare the open field test and light-dark transition test data between the groups. *P* < 0.05 was considered significant for all tests. We used Pearson’s product-moment correlation analysis to examine the relationship between the extent of the lesion and each behavioral index in the SRT and SNT.

## Results

### Social recognition test (SRT) in PL-lesioned mice

**Fig 2:**
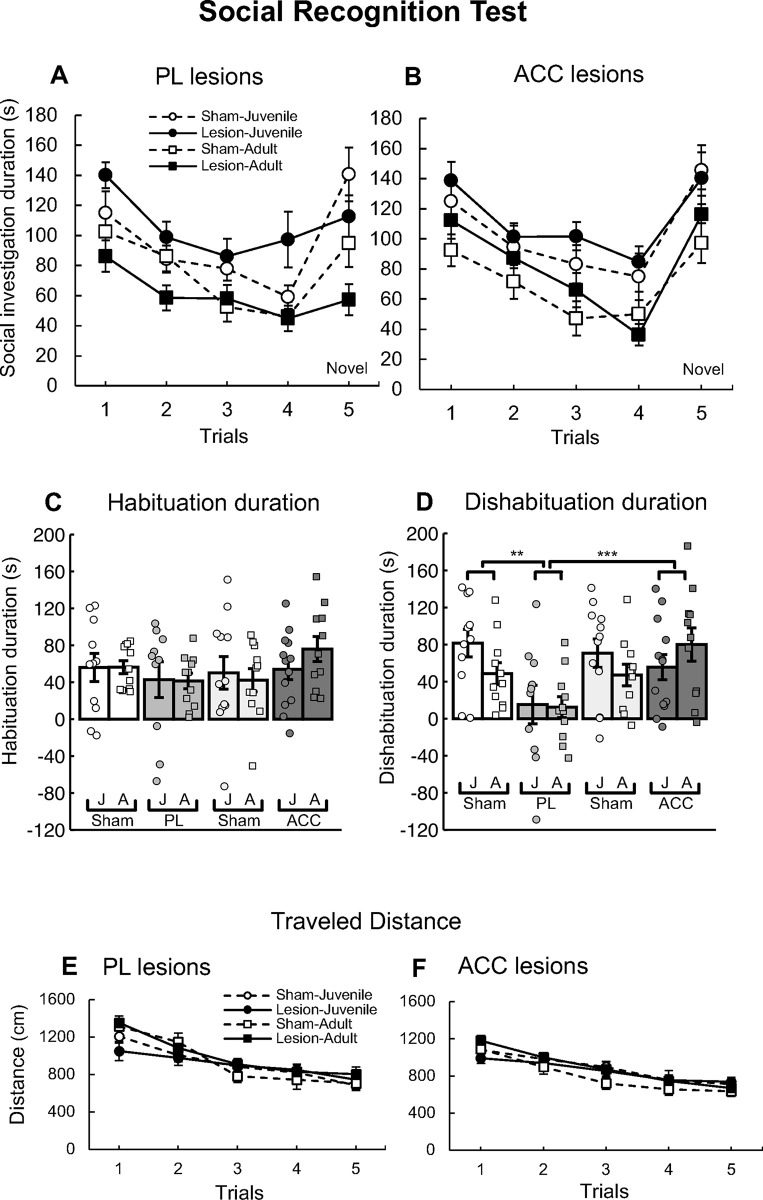
Results of the social recognition test (SRT) in the prelimbic cortex (PL)-lesioned and anterior cingulate cortex (ACC)-lesioned mice. (A) The mean social investigation (SI) duration in the PL lesion group-juvenile (●; *n* = 10), sham control group-juvenile (○; *n* = 11), PL lesion group-adult (■; *n* = 11), and sham control group-adult (□; *n* = 11). (B) The mean SI duration in the ACC lesion group-juvenile (●; *n* = 13), sham control group-juvenile (○; *n* = 12), ACC lesion group-adult (■; *n* = 11), and sham control group-adult (□; *n* = 11). The error bars indicate standard error. (C) The habituation duration (SI duration in the 1st minus that in the 4th trial) and (D) dishabituation duration (SI duration in the 5th minus that in the 4th trial) in the PL and ACC groups. The error bars indicate standard error. The plots indicate the habituation or dishabituation duration for each mouse [J: juvenile stimulus, A: adult stimulus]. The dishabituation duration was shorter in the PL-lesioned group than in the sham group (** *p* < 0.01) and ACC-lesioned group (*** *p* < 0.001). (E, F) The total traveled distance by the PL- (E) and ACC-lesioned mice (F) in the SRT.

Fig 2A shows the duration of SI behavior in the SRT towards the juvenile or adult mouse in the sham control and PL-lesioned groups. Both the sham and lesioned mice spent progressively less time investigating an identical stimulus mouse in the 1st–4th trials. The sham mice but not the lesioned mice spent substantially more time investigating a novel stimulus mouse in the 5th trial. Both the sham and lesioned mice spent more time investigating the juvenile versus adult mice.

A three-way ANOVA for SI duration with lesion (sham, PL-lesioned) × stimulus (juvenile, adult) × trial (1–5) revealed a significant interaction of lesion × trial [*F*(4, 156) = 4.74, *p* = 0.001], main effects of the stimulus [*F*(1, 39) = 14.88, *p* < 0.001], and main effects of the trial [*F*(4, 156) = 22.03, *p* < 0.001]. *Post hoc* analysis (Bonferroni test) revealed that the SI duration in the sham group significantly decreased from the 1st–4th trials (1st > 2nd, 3rd, 4th, *p* < 0.001) and increased in the 5th (5th > 3rd, 4th, *p* < 0.001). In contrast, the SI duration in the PL-lesioned mice significantly decreased from the 1st–4th trials (1st > 2nd, 3rd, 4th, *p* < 0.01 for each comparison) but did not increase in the 5th compared with the 4th trial. Furthermore, a simple effects test revealed that the lesion group had a lower SI duration than the sham group in the 5th trial (*p* = 0.033).

### Social recognition test (SRT) in ACC-lesioned mice

Fig 2B shows the SI duration in the sham control and ACC-lesioned groups for SRT trials with a juvenile or adult mouse. Both the sham and lesioned mice spent progressively less time investigating an identical stimulus mouse in the 1st–4th trials and spent more time investigating a novel stimulus mouse in the 5th trial. Both the sham and lesioned mice spent more time investigating juvenile versus adult mice. A three-way ANOVA for SI duration with lesion × stimulus × trial revealed significant main effects of stimulus [*F*(1, 43) = 10.25, *p* = 0.003] and trial [*F*(4, 172) = 32.74, *p* < 0.001]. *Post hoc* analysis revealed that the SI duration significantly decreased from the 1st through 4th trials (1st > 2nd, 3rd, 4th, *p* < 0.001) and increased in the 5th trial (5th > 2nd, 3rd, 4th, *p* < 0.01).

### Comparison of PL- and ACC-lesioned mice in the social recognition test (SRT)

As shown in Fig 2C, the difference between the SI duration in the 1st and 4th trials in the SRT was calculated as the habituation duration in the PL and ACC groups. All groups had similar habituation durations. A three-way ANOVA for the habituation duration with lesion (lesion, sham) × area (PL, ACC) × stimulus (juvenile, adult) revealed no significant main effects or interactions.

As shown in Fig 2D, the difference between the SI duration in the 5th and 4th trials was calculated as the dishabituation duration in the PL and ACC groups. The PL-lesioned groups had a lower dishabituation duration compared with the other groups. A three-way ANOVA for the dishabituation duration with lesion × area × stimulus revealed a significant interaction of lesion × area [*F*(1, 82) = 8.19, *p* = 0.005], as well as main effects of lesion [*F*(1, 82) = 4.05, *p* = 0.047] and area [*F*(1, 82) = 5.16, *p* = 0.026]. *Post hoc* analysis (the simple effect test) revealed that the dishabituation duration was significantly shorter in the PL-lesioned group compared with the ACC-lesioned (*p* < 0.001) and PL-sham groups (*p* = 0.001).

### Locomotor activity in PL-and ACC-lesioned mice in the social recognition test (SRT)

Fig 2E shows the traveled distance in the SRT for each trial in the PL groups. All groups had a decreased traveled distance across the trials. A three-way ANOVA for traveled distance with lesion (sham, PL-lesioned) × stimulus (juvenile, adult) × trial (1–5) revealed a significant interaction of stimulus × trial [*F*(4, 156) = 3.83, *p* = 0.005] and main effects of the trial [*F*(4, 156) = 52.65, *p* < 0.001].

Fig 2F shows the traveled distance for each trial in the ACC groups in the SRT. All groups had a decreased traveled distance across the trials. A three-way ANOVA for traveled distance with lesion × stimulus × trial revealed a significant interaction of stimulus × trial [*F*(4, 172) = 2.69, *p* = 0.033] and main effects of the trial [*F*(4, 172) = 52.35, *p* < 0.001].

### Social novelty test (SNT) in PL-lesioned mice

**Fig 3:**
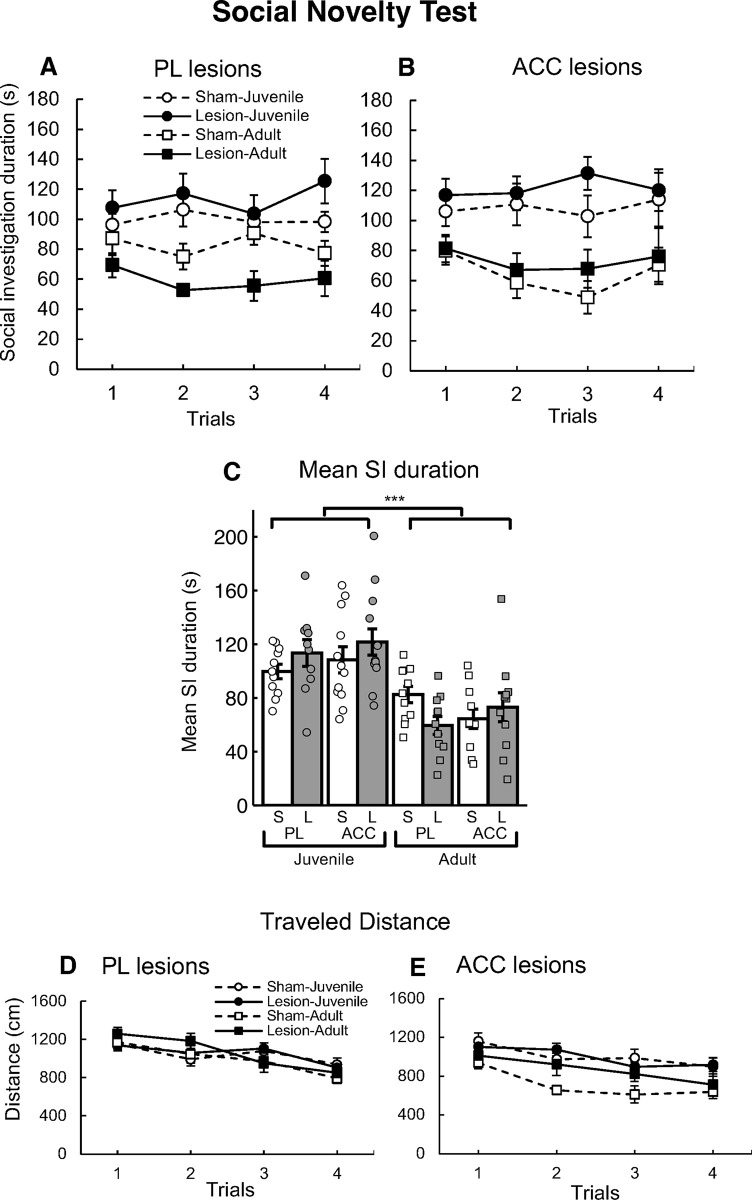
Results of the social recognition test (SNT) in the prelimbic cortex (PL)-lesioned and anterior cingulate cortex (ACC)-lesioned mice. (A) The mean social investigation (SI) duration in the PL lesion group-juvenile (●; *n* = 10), sham control group-juvenile (○; *n* = 11), PL lesion group-adult (■; *n* = 11), and sham control group-adult (□; *n* = 11). (B) The mean SI duration in the ACC lesion group-juvenile (●; *n* = 13), sham control group-juvenile (○; *n* = 12), ACC lesion group-adult (■; *n* = 11), and sham control group-adult (□; *n* = 11). The error bars indicate standard error. (C) The mean SI duration for the four SNT trials in the PL and ACC groups. The error bars indicate standard error. The plots indicate the mean SI duration for each mouse [S: sham control, L: lesion]. The mean SI duration was shorter toward adult versus juvenile stimuli (*** *p* < 0.001). (D, E) Results of the total traveled distance in the PL- (D) and ACC-lesioned mice (E) in the SNT.

Fig 3A shows the SI duration in the SNT in the sham control and PL-lesioned groups. Compared with the control mice, PL-lesioned mice had a shorter SI duration in trials with the adult stimulus. A three-way ANOVA for SI duration with lesion (sham, PL-lesioned) × stimulus (juvenile, adult) × trial (1–4) revealed a significant interaction of lesion × stimulus [*F*(1, 39) = 6.72, *p* = 0.013] and main effects of stimulus [*F*(1, 39) = 25.10, *p* < 0.001]. *Post hoc* analysis (simple effects test) revealed that the SI duration in the PL-lesioned group for trials with an adult stimulus was significantly shorter than that in the sham group [*F*(1, 20) = 6.52, *p* = 0.019], and that the SI duration for trials with a juvenile mouse was significantly longer than that in trials with an adult mouse in both the sham [*F*(1, 20) = 4.46, *p* = 0.048] and lesion groups [*F*(1, 19) = 20.98, *p* < 0.001].

### Social novelty test (SNT) in ACC-lesioned mice

Fig 3B shows the SI duration in the SNT for the sham control and ACC-lesioned mice. Both the sham and lesioned mice spent more time investigating the juvenile versus adult mice. A three-way ANOVA for SI duration with lesion × stimulus × trial revealed significant main effects of stimulus [*F*(1, 43) = 23.41, *p* < 0.001].

### Comparison of PL- and ACC-lesioned mice in the social novelty test (SNT)

Fig 3C shows the mean SI duration for the four SNT trials in the PL and ACC groups. The mean SI duration toward the adult stimulus was less than that toward the juvenile stimulus. A three-way ANOVA for the mean SI duration with lesion (lesion, sham) × area (PL, ACC) × stimulus (juvenile, adult) revealed significant main effects of stimulus [*F*(1, 82) = 45.82, *p* < 0.001].

### Locomotor activity of PL- and ACC- lesioned mice in the social novelty test (SNT)

Fig 3D shows the traveled distance for each trial in the SNT. All groups had a decreased traveled distance across the trials. A three-way ANOVA for traveled distance with lesion (sham, PL-lesioned) × stimulus (juvenile, adult) × trial (1–4) revealed a significant interaction of stimulus × trial [*F*(3, 117) = 4.83, *p* = 0.003] and the main effects of the trial [*F*(3, 117) = 45.17, *p* < 0.001].

Fig 3E shows the traveled distance for each trial in the ACC groups in the SNT. All groups had a decreased distance traveled across the trials. A three-way ANOVA for traveled distance with lesion × stimulus × trial revealed significant main effects of the stimulus [*F*(1, 43) = 10.51, *p* = 0.002] and trial [*F*(3, 129) = 18.96, *p* < 0.001].

### Open-field test in PL- and ACC-lesioned mice

**Fig 4:**
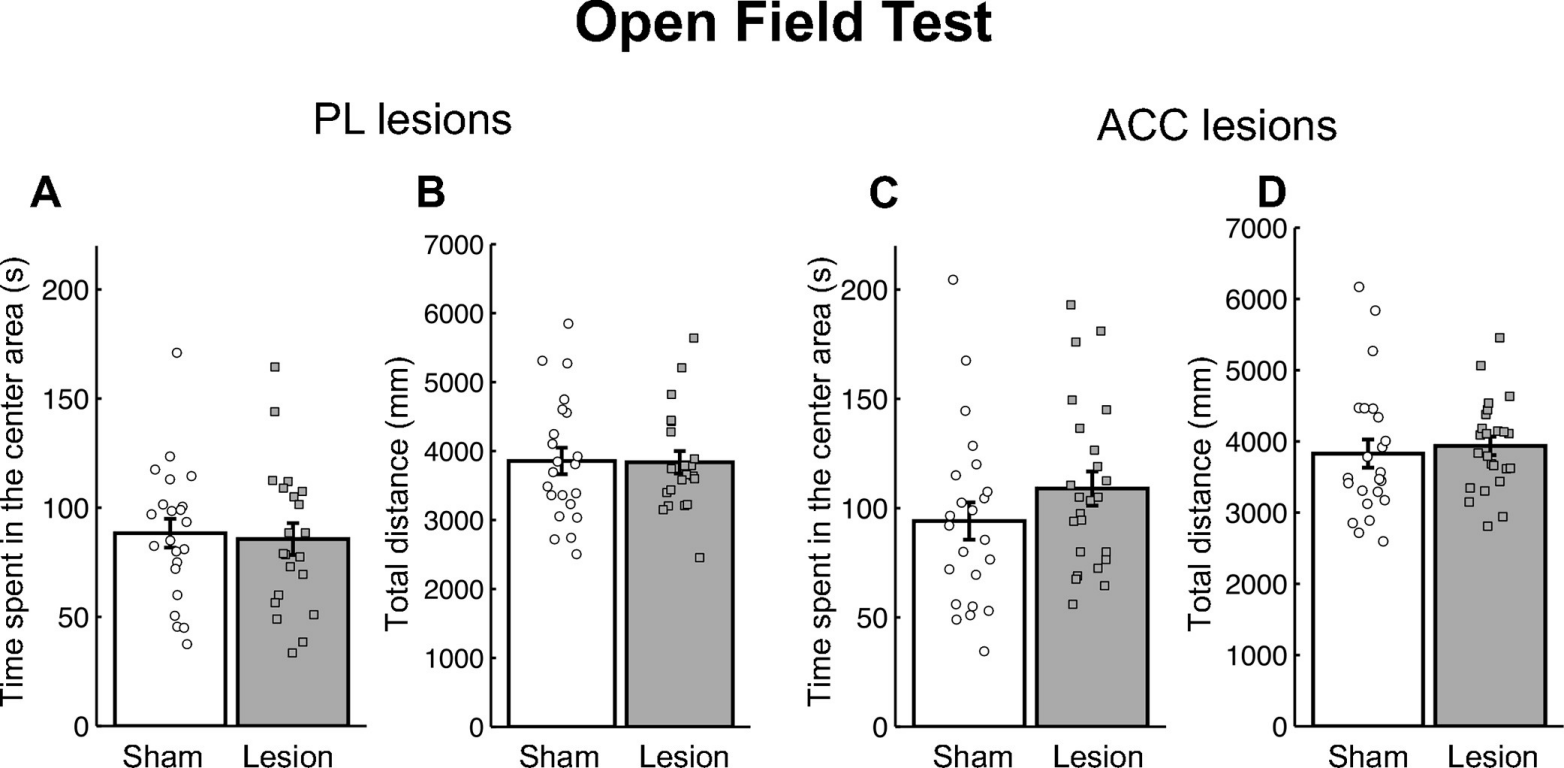
Results of the open field test in the prelimbic cortex (PL)- (A, B) and anterior cingulate cortex (ACC)- (C, D) lesioned mice. Time spent in the center area (A, C) and total distance traveled (B, D). The error bars indicate standard error. [PL sham group (*n* = 22), PL lesion group (*n* = 21), ACC sham group (*n* = 23), ACC lesion group (*n* = 24)].

Fig 4A and 4B show the total distance traveled and the time spent in the center area in the open field test for the sham and PL-lesioned groups. A Student’s *t*-test revealed no significant differences in the total distance traveled [*t*(41) = 0.07, *p* = 0.941] or the time spent in the center area [*t*(41) = 0.27, *p* = 0.786] between the sham and PL-lesioned groups. Fig 4C and 4D show the total distance traveled and the time spent in the center area in the sham and ACC-lesioned groups. A Student’s *t*-test revealed no significant differences in the total distance traveled [*t*(45) = 0.46, *p* = 0.647] or time spent in the center area [*t*(45) = 1.29, *p* = 0.204] between the sham and ACC-lesioned groups.

### Light-dark transition test in PL- and ACC-lesioned mice

**Fig 5:**
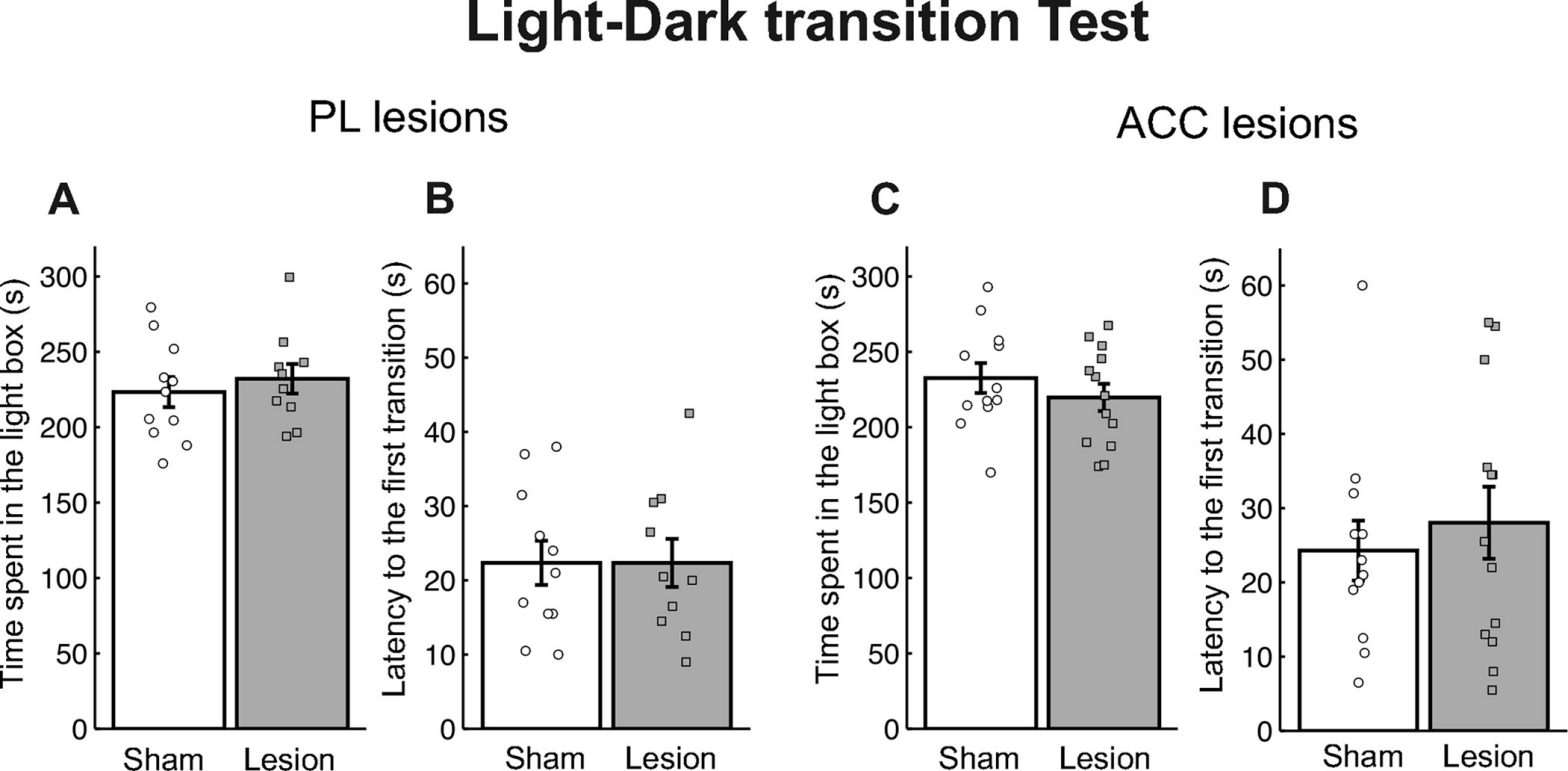
Results of the light-dark transition test in the prelimbic cortex (PL)- (A, B) and anterior cingulate cortex (ACC)- (C, D) lesioned mice. Time spent in the light box (A, C) and latency to the first entry into the light box (B, D). The error bars indicate standard error [PL sham group (*n* = 11), PL lesion group (*n* = 10), ACC sham group (*n* = 12), ACC lesion group (*n* = 13)].

Fig 5 shows the time spent in the light box and the latency to the first entry into the light box in the light-dark transition test for the PL-lesioned (Fig 5A and 5B) and ACC-lesioned (Fig 5C and 5D) mice. A Student’s *t*-test revealed no significant differences in the time spent in the light box [*t*(19) = 0.63, *p* = 0.537] or latency of first entry into the light box [*t*(19) = 0.00, *p* = 0.998] between the sham and PL-lesioned mice. Similarly, a Student’s *t*-test revealed no significant differences in the time spent in the light box [*t*(23) = 0.96, *p* = 0.346] or latency of first entry into the light box [*t*(23) = 0.59, *p* = 0.562] between the sham and ACC-lesioned mice.

### Histology

**Fig 6:**
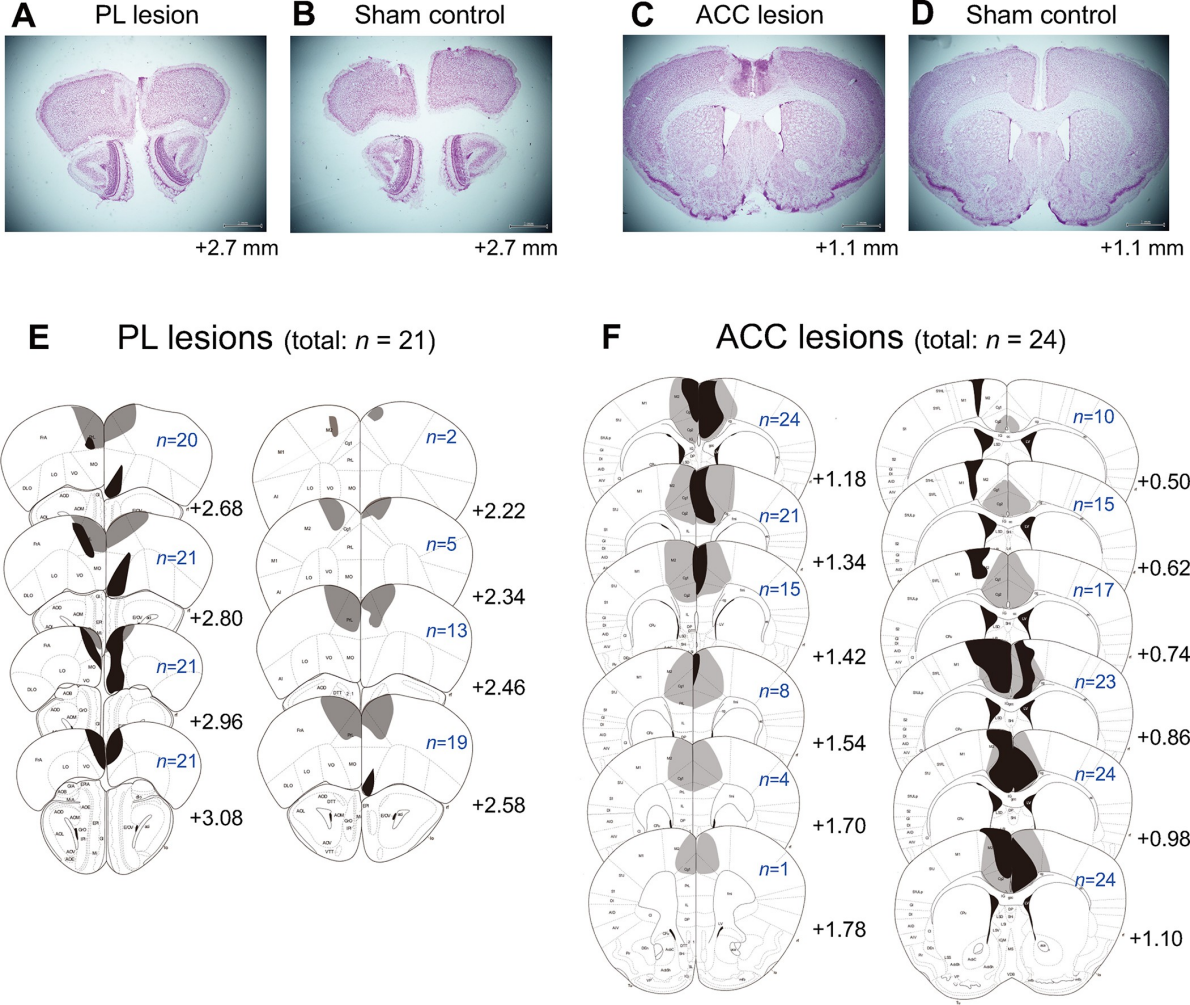
Representative brain sections from the prelimbic cortex (PL)-lesioned (A) and sham control mice (B), and the maximum (light gray areas) and minimum (dark gray shaded areas) extent of cell loss in PL-lesioned mice (E). All PL-lesioned mice exhibited injury in the PL and frontal association cortex. Representative brain sections from the anterior cingulate cortex (ACC)-lesioned (C) and sham control mice (D), and the maximum (light gray areas) and minimum (dark gray shaded areas) extent of cell loss in ACC-lesioned mice (F). All ACC-lesioned mice exhibited injury in the ventral and dorsal ACC and secondary motor cortex. The black numbers indicate the anteroposterior distance (mm) of the sections relative to the bregma. The blue numbers indicate the number of animals for which the brain sections were lesioned. All scales indicate 1 mm.

The PL or ACC lesions were verified after the behavioral tests were complete. Fig 6 shows representative brain sections from PL-lesioned (Fig 6A) and sham mice (Fig 6B) and from ACC-lesioned (Fig 6C) and sham mice (Fig 6D). Fig 6 shows the maximum (light gray shaded) and minimum (dark gray shaded) extent of neuronal cell loss in the PL- (Fig 6E) or ACC- (Fig 6F) lesioned groups. In the PL-lesioned mice, the PL was severely injured and the medial parts of the frontal pole were also slightly damaged. Some PL-lesioned mice exhibited slight injuries in the secondary motor cortex, orbitofrontal cortex, or anterior part of the ACC. In the ACC-lesioned mice, the ventral and dorsal ACC and medial parts of the secondary motor cortex were severely injured. Some ACC-lesioned mice exhibited slight injury in the first motor cortex and the posterior part of the PL. The general activity of mice (*n* = 6) with a partially injured first motor cortex was not different from that of the other ACC-lesioned mice (*n* = 18) in the open-field test [*t*(22) = 0.07, *p* = 0.944]. No injuries other than needle traces were observed in all sham mice.

**Fig 7:**
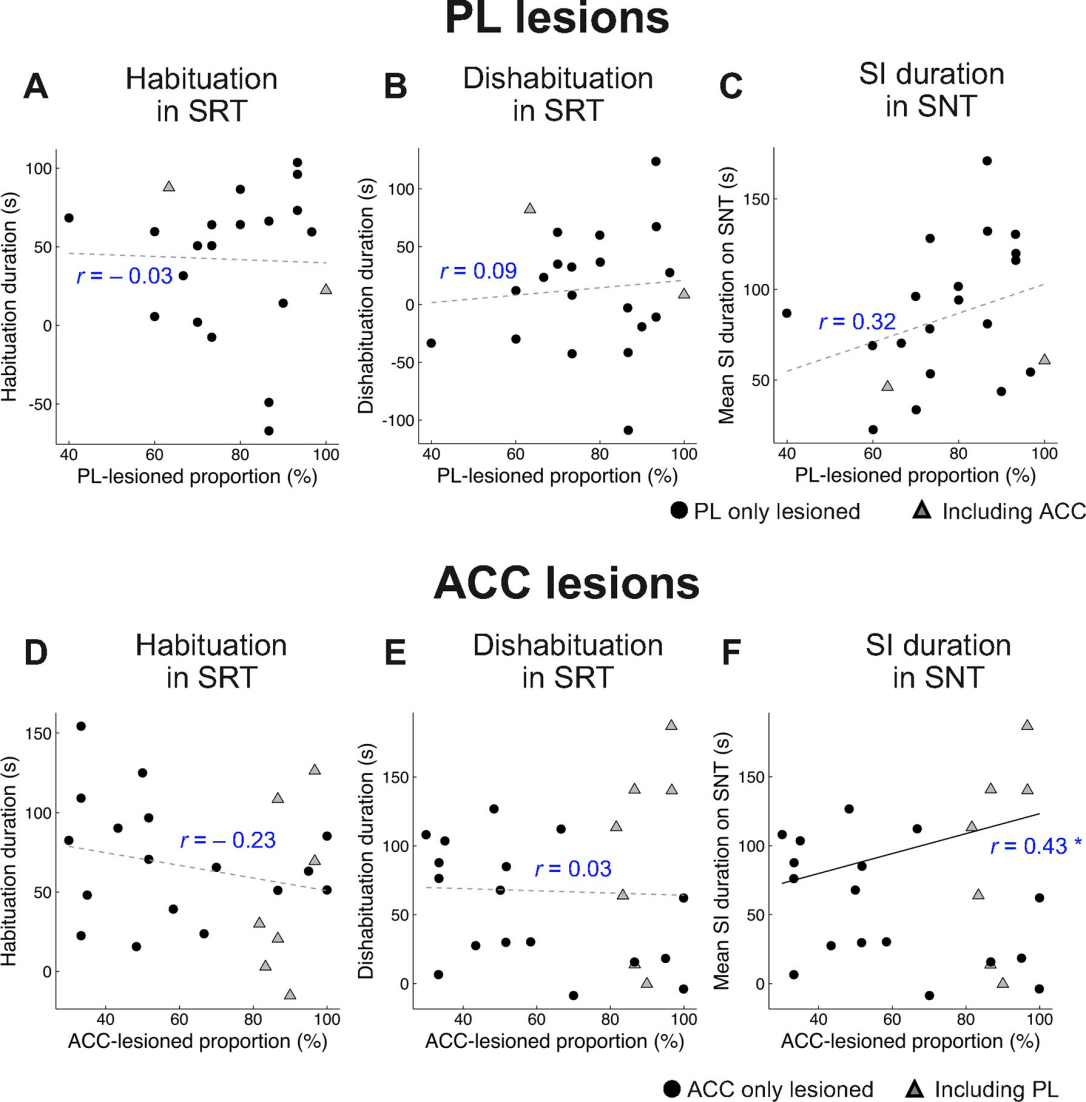
Scatter plots showing the extent of the prelimbic cortex (PL) or anterior cingulate cortex (ACC) lesions and social investigation (SI) behaviors in the social recognition test (SRT) and social novelty test (SNT). (A, D) Habituation duration and (B, E) dishabituation duration of PL- or ACC-lesioned mice in the SRT. (C, F) Mean SI duration of PL- or ACC-lesioned mice in the SNT. “*r*”indicate Pearson’s product-moment correlation coefficient (* *p* < 0.05).

Fig 7 contains scatter plots showing the extent of the PL or ACC lesions and each index of SI behaviors in the SRT and the SNT. We used Pearson’s product-moment correlation analysis to analyze the relationship between the extent of PL or ACC lesions and the habituation duration in the SRT (Fig 7A and 7D), dishabituation duration in the SRT (Fig 7B and 7E), or mean SI duration in the SNT (Fig 7C and 7F). The results revealed a significant positive correlation (*r* = 0.43, *p* = 0.038) between the extent of the ACC-lesion and the mean SI duration in the SNT (Fig 7F).

## Discussion

### The PL is involved in short-term social recognition and social investigation

We examined the role of the PL in short-term social recognition and social investigation with respect to juvenile or adult male conspecifics. In the SRT, both the sham control and the PL-lesioned mice spent progressively less time investigating an identical stimulus mouse (both juvenile and adult) over the 1st–4th trials. In the 5th trial, the sham mice but not PL-lesioned mice exhibited increased investigation of the novel stimulus mouse (Fig 2A). These results suggest that PL-lesioned mice showed habituation toward the identical stimulus mouse but impaired dishabituation toward the novel stimulus mouse (Fig 2C, 2D). That is, while the PL-lesioned mice recognized the familiar stimulus mouse, they might not have been able to discriminate a novel stimulus from a familiar stimulus. These results are consistent with the recent finding that GABAergic interneurons in the PL play an important role in discriminating novel from familiar stimuli [46]. Furthermore, the PL-lesioned mice in our study exhibited normal habituation, which supports the previous finding that PFC- (PL, IL and ACC) lesioned mice exhibited short-term habituation [19]. These data suggest that habituation and dishabituation (discrimination) processes might involve different brain mechanisms.

In the SNT, the PL-lesioned mice had a significantly shorter SI duration for adult male stimuli compared with the sham control mice (Fig 3A). These results indicate that the PL plays a critical role in approach behavior, that is, this region is involved in social investigation toward adult male mice, but not juveniles. These are new findings regarding the role of the PL in social investigation with respect to adult male conspecifics. Our results are inconsistent with a previous report that PL lesions enhanced social interaction with adult male mice [5]. This discrepancy could be related to differences in the behavioral test procedures. While the previous study measured direct social interactions between the test mouse and the stimulus mouse including those involving physical contact [5], our study indirectly measured SI behavior in the test mice toward the adult stimulus mice in a situation where physical contact was not possible. Thus, our procedure may be superior for evaluating the social investigation of the test mice independent of the social investigation of the stimulus mice [19, 32].

In the SRT, reduced social investigation with respect to the novel adult male in PL-lesioned mice might impair dishabituation in the 5th trial. However, the PL-lesioned mice also showed no dishabituation toward the juvenile mice, although social investigation toward the juvenile mice appeared to be normal. These results indicate that the PL-lesioned mice exhibited impaired social recognition regardless of the type of social stimuli. In addition, we only observed impaired social investigation in PL-lesioned mice toward adult mice.

These comprehensive results highlight the importance of using various social stimuli that induce different types of social investigation, in addition to conducting tests of social recognition and social investigation, when examining the neural mechanisms that underlie social cognitive function. Our data indicate that the PL is critically involved in the social recognition of both juvenile and adult male conspecifics, as well as social investigation towards adult conspecifics. In the SNT, the SI duration in the PL-lesioned mice did not decrease across the four trials. However, our data do not indicate whether the PL-lesioned mice discriminated the four novel stimuli as different mice.

In the SRT and SNT, the distance traveled in the sham and PL-lesioned groups decreased across the trials (Figs 2E and 3D). Our results indicate that all groups of mice were habituated to the experimental situation, and there were no differences in general activity.

### The ACC is not involved in short-term social recognition or social investigation

We also examined the role of the ACC in short-term social recognition memory and social investigation toward juvenile or adult male conspecifics. The sham control and ACC-lesioned mice showed habituation and dishabituation toward both stimuli (Fig 2B–2D), suggesting that the ACC might not play a role in social recognition memory. In the SNT, we found no differences in SI duration between the sham and ACC-lesioned groups throughout the four successive trials (Fig 3B and 3C). These results suggest that the ACC might not play a role in social investigation.

In a previous study, ACC-lesioned rats showed impaired short-term social recognition memory toward juvenile conspecifics and increased social interaction toward adult male conspecifics [6]. This is inconsistent with our findings. This discrepancy might be related to differences in the behavioral tests’ procedures used. While both social recognition memory and social investigation were measured directly via social interactions in the previous study [6], we measured the SI behavior of test mice toward a stimulus mouse in a cylinder. Furthermore, the role of the ACC in social cognitive function might differ between mice and rats because of species-specific cytoarchitectural differentiation [47].

In the SRT and SNT, the distance traveled in the sham and ACC-lesioned groups decreased across the trials (Figs 2F and 3E). Our results indicate that all groups of mice were habituated to the experimental situation, and there were no differences in general activity.

### The PL and ACC may have different roles in social cognitive function

The present study suggests that the PL, but not the ACC, plays an important role in short-term social recognition and social investigation. Many studies have examined social recognition using social discrimination (simultaneous discrimination) paradigms, and found the dorsal hippocampus CA2 [22, 27], ventral hippocampus CA1 [30, 48], medial amygdala [28], BLA [28], and NAc [30] to be critical brain sites. The PL and ACC are connected, and have direct and indirect connections to the above-mentioned subcortical areas. However, the present data suggest that only the PL is critical for short-term social recognition. Recent studies have indicated that the ventral hippocampus is an important site for memory storage regarding familiar conspecifics [30, 48], and that GABAergic input from the ventral hippocampus to the PL regulates discrimination between familiar and novel conspecifics [46, 48]. Taken together with our present results, these data enable us to speculate that the ventral hippocampus (or other regions) might be involved in habituation “memory” processes, while the PL might play important roles in dishabituation “discrimination” processes. Our findings are novel with respect to the habituation-dishabituation (successive discrimination) paradigm. Although we did not find that the ACC played a role in social recognition, this region might be involved in long-term social recognition memory for familiar conspecifics, given the importance of the PFC in long-term social recognition memory [18, 19].

PL-lesioned mice had a significantly decreased SI duration for adult stimuli, and an increased SI duration for juvenile stimuli (although this was not statistically significant). These results are unexpected, and indicate that the PL might be involved in the regulation of social investigation with respect to adult male and juvenile conspecifics in the present study. Both the PL and the ACC have direct connections to both the BLA and NAc [41, 42, 49, 50], and these subcortical areas have contrasting roles in social investigation [7, 34, 51, 52]. The BLA is involved in social anxiety [53] while the NAc is critical for social reward [54, 55]. Neural output from the PL to the BLA and NAc regulates social interaction behavior [7, 56]. Neural input from the BLA to the medial PFC (PL and IL) is related to social investigation [34], and appropriate activation in the PL is necessary for the normal expression of social investigation [57]. In addition, the previous study suggests that the adult male stimulus mouse induces divergent behavior in the test mouse, such as approach behavior and/or avoidance behavior [19]. From these previous findings, we speculate that PL lesions may change the balance of neural networks (including the BLA and NAc) that are important in social approach behavior toward adult male and juvenile stimuli. In contrast, ACC lesions had no effect on social investigation, suggesting that the PL or other regions may uphold the balance of neural networks including the BLA and NAc in ACC-lesioned mice.

Social recognition and social investigation are closely linked with the activity of the neuroendocrine system, and are known to influence levels of oxytocin and vasopressin [58, 59]. Previous studies used adult female mice as the test and stimulus mice [32, 40]. As we only used male mice in the present study, future work should examine the role of the PFC and its subregions on social recognition and social investigation in female mice.

### PL and ACC lesions have no effects on general activity or anxiety-related behavior

In the open field test, we found no differences between the sham control and lesion groups in terms of the total distance traveled or the amount of time spent in the center area in both the PL and ACC groups. The amount of time spent in the light box and the latency of the first entry into the light box in the light-dark transition test for the PL- and ACC-lesioned mice were similar to those in the sham mice. These results indicate that the PL and ACC lesions did not affect general activity or anxiety-related behaviors.

Our results are consistent with some previous studies that reported that PL and/or ACC lesions did not affect anxiety-related behaviors in the elevated maze or light-dark transition tests [6, 8, 19, 60]. In contrast, other studies have indicated that inactivation of the medial PFC (PL and IL) or ACC decreased anxiety-related behaviors in the elevated plus maze test [10, 61], and that activation of the PL increased anxiety-related behaviors in the open field test [11]. These data were obtained using different methods, i.e., permanent lesions and pharmacological inactivation of brain areas. Thus, the differences in the results could be related to the acute effects of the drug injection or compensatory action following permanent lesions [62]. Although the PL and ACC may play roles in the modulation of anxiety-related behavior, some subcortical areas (*e*.*g*., the amygdala) appear to be critical for the expression of anxiety-related behaviors [34, 63].

## Conclusion

We used the SRT and SNT to examine the roles of the PL and ACC in short-term social recognition and social investigation. PL-lesioned mice exhibited habituation, but not dishabituation, in the SRT, and showed a lower SI duration for adult male stimuli in the SNT. These results suggest that the PL plays an important role in social recognition of juvenile and adult male conspecifics, and in social investigation for adult male conspecifics only. ACC-lesioned mice did not show impaired short-term social recognition or social investigation for either stimulus. Neither PL lesions nor ACC lesions affected general activity or anxiety-related behavior in the open field test and light-dark transition test. The present data represent new findings regarding the role of the PL in social recognition and social investigation in mice, and contribute to our understanding of the role of the PFC subregions in social cognitive functions.
